# Research progress on the application of nanoparticles delivery in the treatment of atherosclerosis: implications for therapeutic interventions

**DOI:** 10.3389/fcell.2025.1698110

**Published:** 2025-11-10

**Authors:** Menglei Hao, Yaoling Wang, Shaomin Zhang, Shuaishuai Yu, Chunheng Mo, Jinhui Wu

**Affiliations:** 1 Center of Gerontology and Geriatrics, National Clinical Research Center for Geriatrics, West China Hospital, Sichuan University, Chengdu, Sichuan, China; 2 Core Facilities of West China Hospital, Sichuan University, Chengdu, China; 3 Key Laboratory of Birth Defects and Related Diseases of Women and Children of MOE, State Key Laboratory of Biotherapy, West China Second University Hospital, Sichuan University, Chengdu, China

**Keywords:** atherosclerosis, nanoparticles, delivery system, targeted therapy, therapeutic interventions

## Abstract

Atherosclerosis (AS) is a chronic cardiovascular disease and a leading cause of global morbidity and mortality. Its pathological features include lipid accumulation within the arterial walls, macrophage infiltration, and the proliferation of fibrous plaques, which can manifest in various blood vessels throughout the body. The dislodgement of arterial plaques can lead to severe complications, such as myocardial and cerebral infarction. Current therapeutic strategies for AS focused on managing risk factors, including hypertension, and dyslipidemia. However, the adverse effects of medications and the progression of plaques remain challenges. Nanoparticles (NPs), defined as naturally occurring or biosynthesized particles with immunomodulatory properties and sizes ranging from 1 nm to 100 nm, represent a novel drug delivery system. This technology enhances drug stability and targeting specificity while reducing off-target toxicity. NPs encapsulating therapeutic agents or gene-editing components are capable of facilitating transmembrane transport and cross-barrier release, effectively targeting AS plaques to modulate the activity of macrophages and endothelial cells. This mechanism aids in the treatment of plaques and subsequently reduces complications. The advancement of NPs delivery technology offers extensive potential for AS-targeted therapies and personalized medicine. This review aims to summarize recent advances in NP-based delivery systems for AS treatment.

## 1 Introduction

Atherosclerosis (AS) is a chronic cardiovascular disease and a leading cause of global morbidity and mortality. The pathological characteristics of AS include lipid accumulation within the arterial wall, infiltration of macrophages, and the proliferation of fibrous plaques. This condition can manifest in various blood vessels throughout the body, including the coronary arteries, carotid arteries and abdominal aorta. The dislodgement of arterial plaques can lead to severe complications, such as myocardial and cerebral infarction (Libby et al., 2019; Zhaolin et al., 2019; Bonfiglio et al., 2025; Fang et al., 2025a; Zhang et al., 2024). A study of over 30,000 middle-aged individuals revealed 42% had AS, with 5.2% experiencing significant stenosis. Additionally, a retrospective survey indicated that about 20% of middle-aged and elderly people have cervical AS. As the population ages, the number of AS patients is expected to rise (Bergström et al., 2021; Nielsen et al., 2024). AS patients have no obvious symptoms in the early stage, but acute and chronic ischemic changes may occur in the middle and late stages, which seriously affect the quality of life and prognosis (Jones et al., 2021; Bonati et al., 2022). The pathogenesis of AS includes lipid theory, endothelial injury, smooth muscle transformation and inflammation theory. In addition, ferroptosis and dysbiosis are also involved (Engelen et al., 2022; Luqman et al., 2024; Lin et al., 2025; Yan et al., 2024). Current AS treatments focused on managing risk factors like blood pressure, blood sugar, and lipids, stabilizing plaques, and preventing thrombosis. Despite this, many patients still suffer from drug side effects and plaque-related complications (Ward et al., 2019; Li et al., 2023).

Nanoparticles (NPs) are typically defined as naturally occurring or biosynthesized particles with immunomodulatory properties, ranging in size from 1 nm to 100 nm. The technology for delivering NPs represents an innovative drug delivery system that enhances drug stability, and mitigates toxicity and side effects (Lu et al., 2023; Hu et al., 2022). NPs have the capability to alter the stability and solubility of encapsulated compounds, facilitate the transmembrane transport and cross-barrier release of therapeutic agents, and modulate the release site according to therapeutic objectives to achieve efficient delivery. The advancement of NPs delivery offers extensive applications in precision medicine, targeted therapy, and personalized medicine (Mitchell et al., 2021; Jin et al., 2025; Soukar et al., 2025; Cai and Shubhra, 2025). In AS treatment, NPs can deliver statins and other drugs directly to plaques, enhancing drug potency and minimizing systemic distribution. They can also safely transport non-coding RNA for gene editing in plaque cells, enabling targeted reprogramming and treatment (Jiang et al., 2022; Luo Q. et al., 2024; Tang et al., 2024).

A comprehensive literature search was conducted using the PubMed and Web of Science electronic databases for articles published between January 2020 and June 2025. The search employed a combination of the following keywords and their synonyms: “nanoparticle”, “drug delivery”, “atherosclerosis”, “plaque”, “macrophage”, “endothelial cell”, “smooth muscle cell”, “targeted therapy”, and “theranostic”. Relevant articles were selected based on their focus on the application of nanoparticle technology in the context of atherosclerosis pathophysiology and treatment. This review aimed to critically evaluate the advances in NPs delivery, specifically focusing on its application in the treatment of AS.

## 2 Advances in the pathogenesis and treatment of AS

### 2.1 Pathogenesis of AS

The pathogenesis of AS is multifaceted, with dyslipidemia being a primary initiating factor. Elevated low-density lipoprotein (LDL) cholesterol can penetrate and oxidize within damaged vascular endothelium. Macrophages consume these oxidized lipids, forming foam cells that create the plaque’s lipid core. AS is initiated by endothelial damage, blood lipids, and toxins. Cholesterol deposits in arterial walls encourage foam cell growth and plaque development. Additionally, during inflammation, arterial SMCs can infiltrate the intima, absorbing LDL and becoming foam cells (Libby et al., 2011; Tamargo et al., 2023; Dong et al., 2025). Genetic and external factors like aging, hypertension, diabetes, obesity, and smoking can lead to metabolic disorders that damage vascular endothelial cells, increasing the risk and progression of AS (Fang et al., 2025a). These cells then secrete fewer relaxing and more contractile factors, limiting vascular relaxation and promoting platelet aggregation, which creates a thrombotic microenvironment. Aging also causes epigenetic changes, cell damage, and oxidative stress, further contributing to AS (Wang and Bennett, 2012; Smit et al., 2023).

Diabetic patients experience heightened vascular inflammation and oxidative stress, leading to increased aerobic glycolysis in endothelial cells. This damage attracts inflammatory cells, and activates inflammatory pathways. Persistent reactive oxygen species (ROS) and oxidative stress further collaborate with vascular inflammation to aggravate plaque formation (La Sala et al., 2019; Yuan et al., 2019). Obesity increases the risk of insulin resistance, hyperlipidemia, and microbiota imbalance, as well as metabolic disorders, and oxidative stress. This leads to immune cell infiltration and pro-inflammatory factor release, accelerating plaque progression (Rocha and Libby, 2009; Qiao et al., 2022; Zhou D. et al., 2022). Overall, the key target cells for atherosclerosis treatment include ECs, macrophages, smooth muscle cells (SMCs), and foam cells.

### 2.2 Current treatments for AS

AS treatment involves managing risk factors like blood pressure, glucose, lipids, and stabilizing plaques. Long-term statin use can greatly lower LDL cholesterol and stabilize plaques (Nelson et al., 2022). Antiplatelet drugs like aspirin and clopidogrel prevent platelet clumping, thrombosis, and lower the risk of strokes. Anti-inflammatory and antioxidant therapies reduce vascular inflammation, prevent LDL oxidation, improve ECs function, and delay atherosclerosis by inhibiting foam cells formation (Gawaz et al., 2023; Malekmohammad et al., 2019). Traditional Chinese medicine (TCM) interventions have shown potential in regulating lipid metabolism, attenuating vascular inflammation, and stabilizing atherosclerotic plaques. For instance, Tongxinluo capsules have been demonstrated to protect vascular endothelial function, inhibit vascular SMCs proliferation, and enhance plaque stability (Jiang et al., 2023). Bioactive TCM compounds, including berberine, curcumin, and ginsenosides, exhibit multi-faceted therapeutic potential against atherosclerosis through their anti-inflammatory, antioxidant, and lipid-regulating properties. Harnessing NP-based delivery systems can overcome their inherent pharmacokinetic limitations, enhancing bioavailability, targeting precision, and safety profiles. For instance, curcumin-loaded NPs potently suppress inflammatory signaling in macrophages and ECs, attenuating AS progression (Zhao et al., 2020; Liu et al., 2020; Fontana et al., 2024). Advancing TCM-inspired nanomedicines represents a promising frontier for developing precise and personalized AS therapies.

In addition, the drug PCSK9 inhibitor specifically binds to the PCSK9 protein, prevents the degradation of low-density lipoprotein receptors, lowers cholesterol levels, and improves oxidative stress and inflammation homeostasis. It is suitable for patients with refractory hyperlipidemia and some statin-tolerant patients (Punch et al., 2022; Shin et al., 2024). AS causes severe stenosis of the vascular lumen and symptoms require surgical intervention to improve ischemia, including coronary artery bypass grafting, percutaneous coronary intervention, and carotid endarterectomy (Firnhaber and Powell, 2022). However, current therapeutic strategies have significant limitations. Lipid-lowering agents, particularly statins, have been associated with adverse effects including hepatic dysfunction and clinical symptoms such as myalgia, muscle weakness, and constipation. Antiplatelet therapies carry inherent risks of hemorrhage, while surgical stents, being foreign bodies, may induce thrombosis and inflammatory responses, in addition to incurring substantial medical expenses. Consequently, there are urgently needed for more efficacious and targeted treatment strategies for AS (Cha et al., 2025; Gurbel et al., 2019).

## 3 Current application of NPs delivery technology

### 3.1 NPs delivery of therapeutic agents

NPs achieve precise delivery of therapeutic agents, including small-molecule drugs and nucleic acids (e.g., miRNAs), through encapsulation or surface modification. This approach holds significant potential for application across a range of diseases. For instance, Guo et al. developed a nanoparticle-based miR-21-3p delivery system to transport miRNA molecules to tumor cells, regulating lipid peroxidation and interferon-γ-induced ferroptosis, thereby synergizing with anti-PD-1 immunotherapy in melanoma (Guo et al., 2022; Xu J. et al., 2023; Zhou A. et al., 2024; Yan et al., 2025).

The capability of NPs to deliver bioactive molecules to dysfunctional ECs is a key therapeutic principle. For instance, NPs-mediated delivery of the transcription factor FOXM1 has been demonstrated to promote endothelial regeneration in models of vascular injury, a mechanism directly relevant to restoring endothelial homeostasis in atherosclerosis. Similarly, the strategy of using macrophage-targeted NPs to deliver anti-inflammatory agents (e.g., miRNA-146a) and modulate macrophage phenotype is directly translatable to dampening macrophage-driven inflammation in atherosclerotic plaques (Huang et al., 2023; Fei et al., 2023). Overall, NPs delivery can induce therapeutic agents release according to therapeutic purposes.

### 3.2 Polymer NPs delivery

The delivery of mRNA based on polymer NPs can carry modified photosensitizers and release drugs under the induced light of specific wavelengths, thus constructing a ROS-responsive polymer NPs platform (Zhou et al., 2023). Similarly, NPs can deliver drugs across the blood-brain barrier to reach the central nervous system. The particle polymer slowly releases the drugs encapsulated in NPs under ultrasound induction, which can reduce the level of pro-inflammatory factors and inhibit neuroinflammation (Jiang et al., 2024). NPs can be synergistically administered alongside cells and small molecule regulators to achieve specific therapeutic objectives. Furthermore, NPs can be co-administered with cells (e.g., mesenchymal stem cells) to achieve synergistic therapeutic effects, as shown in models of tissue injury and repair (Hua et al., 2024).

### 3.3 NPs as platforms for innovative therapeutic delivery

Beyond conventional routes, NPs serve as versatile platforms for innovative therapeutic modalities, addressing key limitations of current treatments. A prominent application lies in enhancing patient compliance and bioavailability. For instance, NPs can function as carriers for biologics like insulin, enabling non-invasive oral or nasal administration and thereby overcoming the drawbacks of subcutaneous injections, such as pain and infection (Souto et al., 2019). Similarly, for nucleic acid therapeutics, lipid NPs encapsulating siRNA have been shown to durably maintain glucose homeostasis in diabetic mice, demonstrating the ability of NPs to extend the therapeutic duration of labile molecules (Neumann et al., 2017).

More fundamentally, NP technology enables precise spatial control over therapeutic action, from the organelle to the cellular level (He M. et al., 2024). Zhang et al. exemplified organ-level targeting by developing NPs that efficiently deliver gene-editing machinery to the vascular endothelium, achieving widespread and potent gene knockdown after a single systemic administration. Extending this precision to subcellular compartments, mitochondrially-targeted NPs have been engineered to locally mitigate oxidative stress—a core pathogenic mechanism in cardiovascular diseases—thereby improving pharmacokinetics and minimizing off-target toxicity (Forini et al., 2020; Cai et al., 2024; Zhang et al., 2022; Zheng et al., 2024).

**FIGURE 1:**
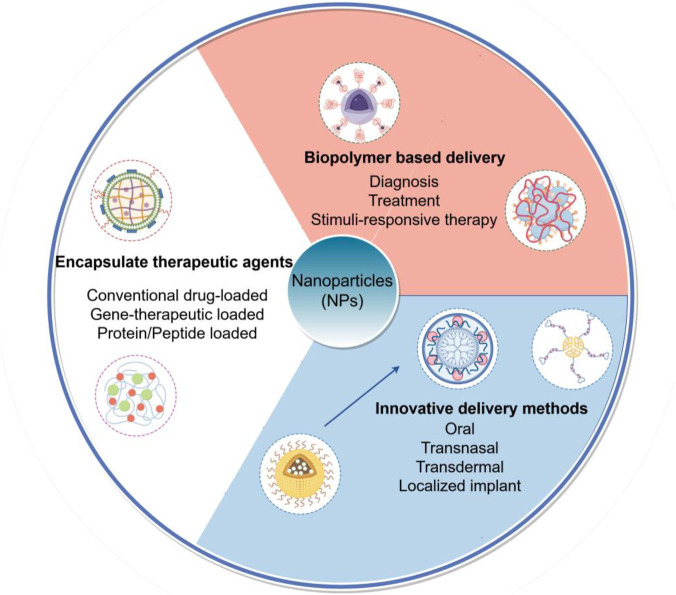
Schematic illustration of multifunctional nanoparticle (NP) platforms for targeted atherosclerosis therapy. This figure outlines the primary strategic paradigms for NP-based interventions in atherosclerosis, categorized by their core design and function: Encapsulation of therapeutic agents, Biopolymer-based delivery systems and Innovative delivery methods.

These diverse examples collectively underscore the transformative potential of NPs as a unified platform technology. They facilitate everything from non-invasive delivery and prolonged efficacy of established drugs to the precise targeting of genes and organelles for next-generation therapies, all of which are highly relevant for advancing the treatment of complex diseases like atherosclerosis (Figure 1).

## 4 The pathophysiological of NPs treatment for AS

### 4.1 NPs delivery modulates AS inflammation

Local inflammation is a pivotal driver of atherosclerotic plaque progression and instability. NPs technology offers sophisticated strategies to precisely deliver anti-inflammatory agents to the plaque microenvironment. A key approach involves the delivery of nucleic acids encoding anti-inflammatory cytokines. For instance, Gao et al. developed polymeric NPs loaded with mRNA encoding the anti-inflammatory cytokine IL-10. Upon targeted delivery to AS plaques, these NPs enable the localized translation and secretion of IL-10 by lesional cells. This targeted IL-10 expression was shown to reprogram macrophages towards an anti-inflammatory M2 phenotype, suppress oxidative stress, and enhance features of plaque stability in ApoE^−/−^ mice (Gao et al., 2023). This mRNA-based strategy allows for sustained local protein production, overcoming the short half-life of recombinant cytokine proteins.

Biomimetic NPs, cloaked with cell membranes, represent another powerful strategy for active targeting. Li et al. engineered colchicine-loaded NPs coated with macrophage membranes. This biomimetic design, displaying CD47 and integrin α4/β1 from the source cells, conferred dual advantages. The marker CD47 minimized unwanted phagocytic clearance by immune cells, while integrin α4/β1 mediated specific adhesion to activated vascular cell adhesion molecule-1 (VCAM-1)-positive ECs at inflammatory sites. This targeted delivery of colchicine significantly attenuated local inflammation and inhibited AS progression (Li et al., 2022). Expanding on this concept, Huang et al. developed monocyte membrane-encapsulated NPs as a targeted delivery system to activated and inflamed endothelium. These NPs efficiently accumulated on the plaque surface and delivered a YAP/TAZ inhibitor. The treatment effectively suppressed the pro-inflammatory YAP/TAZ signaling axis specifically in AS-prone areas, thereby reducing ECs activation and macrophage infiltration without the risks associated with systemic inhibition of these critical transcriptional regulators (Huang et al., 2024).

In addition, platelet membrane-biomimetic NPs loaded with curcumin have been designed to evade immune cell phagocytosis and improve particle-cell compatibility. These NPs demonstrated enhanced targeting to activated endothelium and potently inhibited the activation of the NF-κB inflammatory pathway, leading to a significant reduction in pro-inflammatory cytokine production and atherosclerotic lesion size (Fontana et al., 2024). Collectively, these studies underscore that the targeted delivery of NPs to macrophages or ECs within the core of atherosclerotic plaques markedly enhances the efficacy of anti-inflammatory pharmacotherapy. Moreover, NPs engineered with endogenous cell membranes hold significant potential for modulating the atherosclerotic immune microenvironment and mitigating local plaque inflammation.

### 4.2 NPs target the functional fate of hub cells

Vascular ECs form the critical barrier between the blood and the vessel wall. In AS, risk factors induce EC dysfunction, characterized by increased expression of adhesion molecules, enhanced permeability, and a shift towards a pro-inflammatory and pro-oxidant state, which initiates and perpetuates atherogenesis (Hu et al., 2023; Qiu et al., 2023; Chen Y. et al., 2024; Qiu Y. et al., 2024). Therefore, NPs designed to restore EC homeostasis represent a foundational therapeutic strategy. NPs can target ECs, inhibit vascular inflammation and reverse dysfunction. Oxidative stress exacerbates the disruption of the ECs barrier. Endothelial-mesenchymal transition (EndoMT) enables ECs to acquire mesenchymal phenotypes and accelerates plaque progression (Xiao et al., 2021; Jiang et al., 2025; Zhou et al., 2025; Fang Z. et al., 2024). A promising strategy involves functionalizing the surface of NPs with membranes derived from ECs. This biomimetic coating can achieve “homologous” or homotypic targeting, as the membrane proteins on the NPs surface retain their natural affinity for ligand-receptor interactions with other ECs, thereby enhancing the accumulation of NPs at atherosclerotic lesions. In the acidic microenvironment, NPs simulate the laminar shear stress effect, activate anti-inflammatory signals, inhibit the inflammatory cascade and thereby suppress plaques. NPs promotes the expression of eNOS and restores vasodilation function. NPs targets the N-cadherin protein of mesenchymal ECs, reverses the EndoMT process, effectively repairs the vascular barrier and inhibits leukocyte extravasation (Bizeau et al., 2017; Yeh et al., 2022; Nasr et al., 2024; Wei et al., 2024; Fang X. et al., 2024).

Vascular SMCs are crucial for plaque stability. However, in AS, they undergo a pathological phenotypic switch from a contractile to a synthetic/pro-inflammatory state, migrating to the intima, secreting extracellular matrix, and contributing to plaque growth and instability (Basatemur et al., 2019; Yin et al., 2024). Targeting this phenotypic transition with NPs is a promising approach to halt plaque progression. NPs targeting SMCs can inhibit phenotypic transformation. SMCs undergo a pathological phenotypic transformation from contractile to synthetic in AS. This process leads to the migration of SMCs to the intima layer and abnormal proliferation, secreting a large amount of extracellular matrix. At the same time, they take up oxidized low-density lipoprotein (ox-LDL) and convert it into foam cells, ultimately promoting the fibrous cap and the formation of unstable plaques. NPs can directionally arrange SMCs through the contact guiding effect, inhibiting their migration and proliferation (Hamczyk et al., 2024; Huang et al., 2022a). The NPs targeted delivery system targets the surface receptors of SMCs through surface modification. NPs can also precisely deliver antiproliferative drugs, inhibit the mTOR pathway, and simultaneously upregulate the expression of contractile markers. In addition, NPs may have both antioxidant and gene regulatory functions. They can eliminate ROS and block ROS-mediated proliferative signals, thereby inhibiting the transformation of SMCs into synchromes (Lu et al., 2018; Huynh et al., 2022).

Macrophages are the central orchestrators of inflammation within atherosclerotic plaques. They internalize modified lipids to become foam cells, secrete pro-inflammatory cytokines, and release proteases that weaken the fibrous cap, making them a prime therapeutic target for NP-mediated intervention (Lin et al., 2021; Fang et al., 2025b). To enhance targeted delivery, NPs can be engineered with ligands that recognize receptors highly expressed on plaque macrophages. For instance, Ni et al. designed reactive oxygen species (ROS)-responsive NPs loaded with small interfering RNA (siRNA) targeting the olfactory receptor Olfr2 in macrophages. Upon accumulation in the atherosclerotic milieu, these NPs released the siRNA, leading to the downregulation of Olfr2. This inhibition subsequently suppressed the activation of the NLRP3 inflammasome and the secretion of interleukin-1β, thereby reducing foam cell formation and delaying plaque progression in mice (Ni et al., 2024).

Beyond gene silencing, NP platforms can be designed to directly modulate critical cellular functions. Qiu et al. developed a biomimetic delivery system by coating NPs with membranes from engineered cells overexpressing the MerTK receptor. When administered to diabetic ApoE^−/−^ mice, these NPs specifically homed to atherosclerotic plaques and were internalized by lesional macrophages. This delivery significantly enhanced the efferocytic (cell-clearing) capacity of macrophages, leading to a reduction in apoptotic cell accumulation, attenuated inflammation, and decreased foam cell formation, thereby stabilizing plaques (Qiu S. et al., 2024). Furthermore, the therapeutic scope of NPs extends to the targeted elimination of senescent cells, a emerging driver of plaque instability. Zhang et al. constructed magnetic NPs conjugated with pro-apoptotic gene plasmids. These NPs were actively guided to atherosclerotic plaques using an external magnetic field. Upon localization, the NPs facilitated the transfection of senescent cells within the plaque, triggering their selective apoptosis. This targeted clearance of senescent cells inhibited plaque progression in ApoE^−/−^ mice with minimal off-target effects, highlighting a novel application of NPs in managing AS (Zhang et al., 2023).

For efficient intracellular delivery, the surface functionalization of NPs is crucial. Studies have shown that by recognizing the stabilin-2 receptor overexpressed by plaque macrophages via S2P polypeptides, NP accumulation can be significantly enhanced. Some of these innovative NP vectors are engineered to possess intrinsic broad-spectrum ROS-scavenging capabilities, which directly inhibit the oxidative stress. The therapeutic cargo they carry, such as inhibitors of folate metabolism, can then simultaneously block the proliferation of macrophages and the secretion of pro-inflammatory factors, creating a multi-pronged therapeutic attack on the inflammatory core of the plaque (Li D. et al., 2024; Akhmedov et al., 2024).

### 4.3 NPs modulates cellular communication

Exosomes are nanoscale extracellular vesicles that facilitate the transfer of proteins and nucleic acids from donor to recipient cells. These vesicles, along with intercellular communication, play a crucial role in the pathogenesis and progression of AS. SMCs exhibit enhanced proliferation and migration capabilities, contributing to the thickening of the arterial wall. In addition, the microRNAs (miRNAs) carried by exosomes can modulate gene expression in target cells, thereby influencing lipid metabolism (Heo and Kang, 2022). The delivery of NPs plays a crucial role in the regulation of gene expression, while exosomes facilitate intercellular communication. Gene therapy utilizing NPs can effectively transport edited genes to vascular ECs, macrophages, and smooth muscle cells, thereby achieving significant regulation of key genes involved in lipid metabolism and rectifying the aberrant expression of genes associated with AS pathogenesis. NPs modified with vascular cell adhesion molecule-1 (VCAM-1)-targeting peptides can specifically bind to activated ECs, while those functionalized with macrophage-specific ligands (e.g., S2P peptide) enhance uptake by plaque macrophages. In addition, Lipid-based NPs were employed to deliver targeted interventions on the lipoprotein-encoding gene LPA, resulting in a substantial reduction in plasma lipoprotein levels and a concomitant decrease of AS (Garcia et al., 2025).

Ni et al. constructed NPs targeting macrophages and downregulated Olfr2 levels, thereby inhibiting the activation of NLRP3 inflammasomes and the secretion of interleukin-1β, thereby inhibiting foam cells and delaying plaque formation (Ni et al., 2024). A notable example of biomimetic precision is the use of neutrophil membrane-encapsulated nanoparticles (NNPs) loaded with antisense oligonucleotides (ASOs) targeting vascular cell adhesion molecule-1 (VCAM-1). This design utilizes the innate tropism of neutrophils for activated endothelium; the neutrophil membrane proteins, particularly CD18, mediate specific adhesion to ECs at atherosclerotic sites, facilitating highly targeted delivery. Once delivered, the ASOs silence VCAM-1 expression, a critical adhesion molecule upregulated on the ECs. This inhibition disrupts the primary step of monocyte adhesion and transmigration into the arterial wall, thereby attenuating the downstream inflammatory cascade and foam cell formation from an upstream, preventative perspective. In addition, the relevance of this neutrophil-mimetic design lies in its superior targeting efficiency and immune evasion compared to earlier systems. Conventional liposomes or polymeric NPs primarily rely on passive accumulation (EPR effect) and lack active, specific targeting motifs, often leading to suboptimal delivery and recognition by the immune system. In contrast, the NNPs’ biomimetic coating not only provides active “homing” capability but also possesses inherent “self” markers that minimize opsonization and clearance by the mononuclear phagocyte system. This combination results in significantly enhanced accumulation at the diseased ECs and more potent therapeutic efficacy than would be achievable with non-biomimetic predecessors (Liu et al., 2023).

**FIGURE 2:**
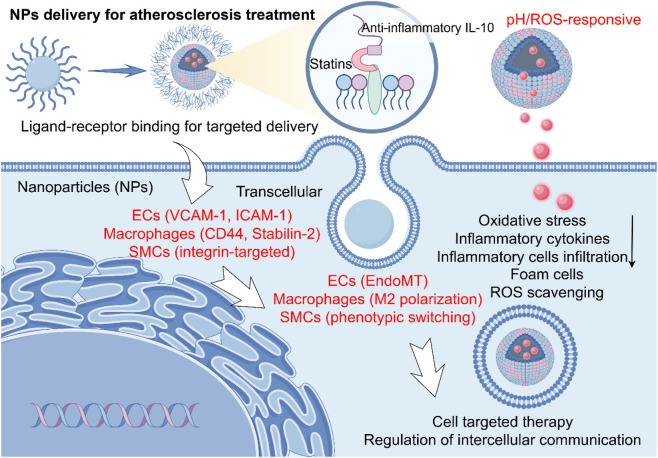
Mechanisms of nanoparticle-mediated therapy in atherosclerosis (AS). The schematic depicts the key cellular players in an atherosclerotic plaque and the targeted therapeutic strategies enabled by nanoparticles (NPs). The NPs modulate key pathological processes—including cellular inflammation, oxidative stress, and foam cell formation—through their distinct interactions with endothelial cells (ECs), macrophages, and smooth muscle cells (SMCs), mediated by specific ligands (e.g., VCAM-1, Stabilin-2, integrins) for targeted delivery, ultimately eliciting therapeutic effects such as Reactive oxygen species (ROS) scavenging and anti-inflammatory signaling.

NPs can also accumulate in macrophages, increase the expression of tyrosine kinase in macrophages at the plaque site, and thus enhance their ability to clear apoptotic cells, reduce inflammation, and the formation of foam cells (Qiu S. et al., 2024). Furthermore, magnetic NPs conjugated with pro-apoptotic gene plasmids can be directed to AS plaques using magnetic field navigation to eliminate senescent cells, thereby inhibiting plaque progression (Zhang et al., 2023). These diverse strategies, which leverage specific targeting and microenvironmental responsiveness, are summarized in Figure 2.

## 5 Applications of NPs delivery in AS

In this section, we provide a detailed overview of recent innovative strategies for nanoparticle-based delivery in atherosclerosis treatment. For each representative study, we specify the type of nanoparticles used, the therapeutic agents loaded, the experimental models employed (including cell lines, animal models, and methods of AS induction), and the key outcomes. Table 1 summarizes these features to facilitate a comparative understanding of the current advances in NPs delivery systems for AS.

**TABLE 1:** Summary of representative nanoparticle-based delivery systems for atherosclerosis treatment.

Category and NP type	Loaded agent(s)	Key targeting mechanism	Target cell/Pathway	Experimental model	Key findings and therapeutic outcome
Macrophage-targeted gene regulation					
S2P-conjugated LNPs *(Cui et al. Circ Res. 2023)*	siRNA (Epsin1/2)	S2P peptide → Stabilin-2 receptor	Lesional macrophages	ApoE^−/−^ mice (WD)	Silenced Epsin, reduced CD36 uptake, enhanced ABCG1 efflux. Plaque regression, decreased necrotic core
S2P-BPNSs *(He et al. Nat Nanotechnol. 2024)*	Resolvin D1	S2P peptide → Stabilin-2 receptor	Macrophages	ApoE^−/−^ mice (HFD)	Potent ROS scavenging, anti-inflammatory, promoted oxidative phosphorylation. Plaque stabilization
Neutrophil-membrane NPs *(Liu et al. ACS Nano. 2023)*	Antisense oligonucleotides	Neutrophil membrane (CD18) → Activated ECs	Endothelial cells (e.g., VCAM-1)	ApoE^−/−^ mice	Targeted gene silencing in plaque endothelium. Attenuated inflammation
Multifunctional and ROS-scavenging NPs					
Dextran-coated polymer NPs (PA/ASePSD) *(Xu et al. Adv Mater. 2023)*	Astaxanthin, SS-31, PMeTPP-MBT	VCAM-1 targeting → Activated ECs/macrophages	Endothelial cells, Macrophages, Mitochondria	ApoE^−/−^ mice	Dual pH/ROS-responsive release. Enhanced M2 polarization, reduced inflammation. Multichannel therapy and imaging
ROS-responsive NPs *(Gao et al. Nat Commun. 2020)*	CCCP (Metabolic modulator)	Not specified (Relies on plaque EPR and microenvironment)	Pro-inflammatory macrophages	ApoE^−/−^ mice (HFD)	Sequestered proinflammatory cytokines, targeted pharmacotherapy. Synergistic anti-inflammatory effect
NCPs *(Lin et al. Small. 2024)*	Simvastatin (ST), Gadolinium	Passive (EPR effect) + pH-responsive release	Plaque microenvironment (Macrophages)	ApoE^−/−^ mice	pH-triggered ST release. Superior plaque reduction and anti-inflammation vs. free ST. Improved targeting and efficacy
Biomimetic and cell-targeted delivery					
Leukosomes *(Boada et al. Circ Res. 2020)*	Rapamycin	Leukocyte membrane proteins (e.g., LFA-1) → Activated ECs (ICAM-1/VCAM-1)	Activated endothelium, Macrophages	ApoE^−/−^ mice (HFD)	Preferential plaque accumulation. Reduced macrophage proliferation and MMP activity. Plaque inflammation reversal
Multifunctional peptide NPs *(Wang et al. J Control Release. 2024)*	Rapamycin	Dual-targeting: subendothelial proteins (e.g., Fibronectin) and ox-LDL	Subendothelial space, Foam cells	ApoE^−/−^ mice	Inhibited platelet adhesion. Reduced foam cell formation and local cytokines. Precise plaque management
Macrophage-membrane NPs *(Li et al. Adv Healthc Mater. 2022)*	Colchicine	Macrophage membrane (CD47) → Immune evasion and targeting	Inflammatory endothelial cells	ApoE^−/−^ mice	Escaped immune clearance, targeted inflamed endothelium. Enhanced anti-inflammatory delivery
Platelet-membrane NPs *(Fontana et al. Adv Healthc Mater. 2024)*	Curcumin	Platelet membrane → Binding to injured vasculature	Activated endothelium, Plaque	ApoE^−/−^ mice (HFD)	Improved biocompatibility, evaded phagocytosis. Suppressed plaque inflammation

Abbreviations: NP, Nanoparticle; AS, Atherosclerosis; ApoE^−/−,^ Apolipoprotein E-deficient; ECs, Endothelial cells; EPR, enhanced permeability and retention; HFD, High-fat diet; LNPs, Lipid nanoparticles; NCPs, Nanoscale coordination polymers; ox-LDL, Oxidized low-density lipoprotein; ROS, reactive oxygen species; WD, western diet.

### 5.1 New strategies for NPs delivery

NPs can be engineered to recognize and actively target the lesion, enabling them to respond to its specific pathological features. The high heterogeneity of AS plaques demands nanocarriers with such precise targeting and responsive capabilities. The new strategy specifically targets the molecular markers overexpressed in the lesion through surface functional modification, such as VCAM-1 and macrophage markers. In addition, the fusion of multiple targeted ligands by NPs or the utilization of the characteristics of the lesion microenvironment can achieve multi-level targeting, significantly enhancing the accumulation efficiency. Stimulation with endogenous oxidative stress markers can further enhance the recognition accuracy and provide a basis for precise intervention (Zou et al., 2024; Wu et al., 2025; Jayakodi et al., 2023; Chen J. et al., 2024). NPs may respond to the microenvironment to trigger controlled-release drugs. For the unique pathological microenvironment within the plaque, such as acidity and high ROS, responsive NPs achieve spatio-temporal controllable drugs release. Acid-base sensitive polymers break in the acidic environment within macrophages. Ros-responsive materials degrade rapidly at oxidative stress sites. In addition, photothermal/photoacoustic response materials can achieve on-demand drugs release and synergistic therapy under near-infrared laser irradiation. The drug release efficiency in response to the conditions increases and significantly reduces off-target toxicity (Todaro et al., 2022; Mukhopadhyay et al., 2024; Zhou X. et al., 2024).

NPs can achieve efficient intracellular delivery and plaque resolution. The core advantage of nanotechnology lies in breaking through cellular barriers to achieve efficient cytoplasmic delivery of therapeutic molecules to key cells such as macrophages and foam cells. Cell-penetrating peptides are delivered through adsorption-mediated delivery. For plaque resolution, NPs co-deliver cholesterol chelating agents to synergistically promote the reverse transport of cholesterol in macrophages. Enzyme-responsive NPs release matrix metalloproteinase inhibitors, which stabilize the plaque fiber caps. NPs encapsulated by specific cell membranes may also actively promote the clearance of apoptotic cells, alleviate local inflammation and enhance plaque regression (Chen et al., 2020; Basak et al., 2022).

NPs may also achieve closed-loop regulation and micro-environment reshaping. The ideal nano-therapy needs to have the ability of dynamic feedback regulation. The intelligent carrier integrates key molecules to monitor drug distribution, carrier degradation and early treatment effects in real time, providing a basis for dose adjustment. The self-regulating system design, such as NPs carrying anti-inflammatory drugs and pro-inflammatory resolution mediators, the inflammatory signal-triggered drug release cascade feedback inhibits the NF-κB pathway and promotes the transformation of macrophages to the repair phenotype. Feedback-type gene vectors enable on-demand biological therapy. Biomimetic materials release vascular endothelial growth factors to promote ECs repair, or secrete extracellular vesicles to regulate immune cell communication. Overall, constructing a robust multi-target feedback network is a key breakthrough point in the future, promoting the development of individualized and dynamically regulated nanotherapy (Dou et al., 2017; Zhou Y. et al., 2022; Peng et al., 2025).

### 5.2 NPs-delivered drugs for AS treatment

Although oral statins are the cornerstone of AS treatment, their efficacy is limited by suboptimal bioavailability and off-target effects. NP-encapsulated targeted drug delivery systems have the potential to enhance therapeutic outcomes. By modifying the surface of liposomal NPs with ligands that precisely recognize specific markers on macrophages within AS plaques, targeted drug delivery can be achieved. This approach allows NPs to transport statins directly to AS plaques, significantly minimizing systemic adverse effects and creating a localized high-concentration drug environment that stabilizes plaque structure and reduces the risk of cardiovascular events. Furthermore, the rupture of AS plaques can precipitate fatal thrombosis, and conventional antiplatelet medications struggle to achieve precise targeting when administered systemically (Li X. et al., 2024).

Polymer NP drug delivery systems can accurately convey antiplatelet agents to vulnerable plaques, and, in response to specific environmental stimuli, they can promptly and precisely release the drugs. This mechanism effectively inhibits platelet aggregation, thereby decreasing the incidence of thrombosis (Luo et al., 2023; Suo et al., 2022). NPs delivered drugs targeting macrophages and endothelial cells can significantly delay the progression of plaques. Cui et al. developed S2P-conjugated lipid nanoparticles loaded with Epsin-specific siRNAs to target lesional macrophages in ApoE^−/−^ mice fed a Western diet. The NPs effectively silenced Epsin expression, leading to reduced CD36-mediated lipid uptake and enhanced ABCG1-dependent cholesterol efflux. This resulted in significant plaque regression, decreased necrotic core area, and improved plaque stability, demonstrating the therapeutic potential of NP-mediated gene silencing in advanced atherosclerosis (Cui et al., 2023).

Macrophage membrane-coated NPs that respond to ROS have been shown to decrease endogenous metabolic clearance, enabling them to target plaques effectively. This targeting reduces local inflammation and inhibits the progression of plaques. In a study by He et al., it was demonstrated that specific nanoparticles can efficiently neutralize a broad range of ROS and suppress the production of atherosclerosis-related proinflammatory cytokines in diseased macrophages. Furthermore, these ROS-responsive modified nanoparticles can deliver lipid-lowering agents to diseased macrophages, thereby further impeding plaque progression (Gao et al., 2020). He et al. developed S2P peptide-modified black phosphorus nanosheets loaded with Resolvin D1 (RvD1), a pro-resolving lipid mediator, for targeted therapy of AS. The NPs exhibited potent ROS-scavenging capability and pH/ROS-responsive drug release. In ApoE^−/−^ mice fed a high-fat diet, RvD1 significantly accumulated in AS plaques and lesional macrophages, leading to reduced plaque area, and suppressed plaque inflammation. Single-cell RNA sequencing further revealed that the treatment downregulated pro-inflammatory pathways and promoted oxidative phosphorylation in macrophages, highlighting a dual-function nanotherapeutic strategy for AS treatment (He Z. et al., 2024).

Xu et al. developed polymer-based nanoplatform (PA/ASePSD) loaded with astaxanthin, SS-31 peptide, and a photoacoustic contrast agent (PMeTPP-MBT). The NPs exhibited dual pH/ROS-responsive drug release and cascade targeting to VCAM-1 on activated ECs and macrophages. The system significantly downregulated CD36 and LOX-1 expression, upregulated ABCA1/ABCG1-mediated cholesterol efflux, and promoted M2 macrophage polarization, leading to reduced plaque burden and inflammation (Xu H. et al., 2023). In a recent study by Lin et al., a pH-responsive theranostic platform based on nanoscale coordination polymers (NCPs) was developed for AS treatment. The NCPs, synthesized with a benzoic-imine linker and Gadolinium (Gd), were coated with a PEGylated lipid bilayer into which simvastatin (ST) was loaded. In an ApoE−/− mouse model of AS, these NPs demonstrated passive targeting to plaques via the enhanced permeability and retention (EPR) effect. Upon encountering the mild acidic microenvironment of the plaque, the NCPs degraded, leading to the controlled release of ST. This targeted delivery resulted in superior anti-inflammatory effects and greater attenuation of plaque progression compared to free ST administration (Lin et al., 2024).

NPs designed for rapamycin delivery have the potential to markedly ameliorate inflammation in AS vascular walls and suppress macrophage proliferation. In ApoE^−/−^ mice, rapamycin delivered via macrophage-mimicking “leukosomes” preferentially accumulated in plaques, potently inhibiting macrophage proliferation and reducing plaque inflammation. Multifunctional peptide-based NPs encapsulating rapamycin, which also inhibit platelet adhesion, demonstrate efficient targeting of subendothelial proteins and oxidized low-density lipoproteins present in plaques. This targeted approach significantly diminishes foam cell formation and local proinflammatory cytokine levels, thereby contributing to the therapeutic management of AS plaques (Boada et al., 2020). A prominent example of innovative targeting is provided by Wang et al., who engineered multifunctional peptide NPs loaded with rapamycin. These NPs were designed to simultaneously target subendothelial proteins (e.g., fibronectin) and oxidized LDL within plaques, while also inhibiting platelet adhesion. The treatment led to a substantial reduction in foam cell formation and local proinflammatory cytokine levels, highlighting its potential for precise plaque management (Wang et al., 2024).

## 6 NPs packaged agents innovations

NPs can achieve efficient targeted delivery of small molecule inhibitors. The clinical translation of traditional small-molecule inhibitors is hampered by poor aqueous solubility, low bioavailability, nonspecific distribution, and systemic toxicity. NPs significantly improve the drug enrichment efficiency at plaque sites by enhancing solubility, prolonging the cycle half-life, and integrating active targeted ligands. NPs, combined with small molecules and triggered by the ROS microenvironment, can significantly increase the concentration of drugs in plaques and significantly inhibit lipid accumulation and inflammation (Luo X. et al., 2024).

NPs can achieve engineered bionic delivery of vesicles. Vesicles constructed by cell membranes and exosomes have good biocompatibility and are easy to functionalize, becoming efficient carriers for the treatment of AS. Through surface modification or direct use of engineered cell-derived vesicles, plaque target and cell penetration ability can be significantly increased. For example, the macrophage cell membrane is coated with NPs, which can efficiently target the activated macrophages/foam cells within the plaque (Yang et al., 2020; Sha et al., 2022).

**FIGURE 3:**
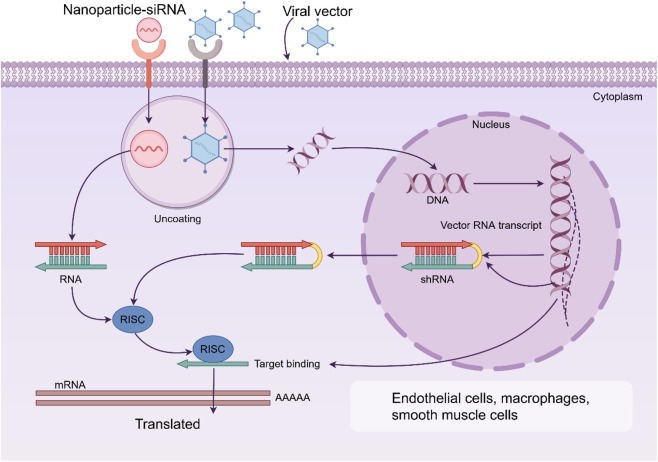
Nanoparticles (NPs) can achieve precise regulation of gene silencing by delivering siRNA. By using the specific receptors highly expressed within the plaques, specific delivery to the endothelial cells, macrophages and smooth muscle cells can be achieved, significantly delaying the progression of the plaques. RISC: RNA-induced silencing complex.

NPs can achieve precise regulation of gene silencing by delivering siRNA. siRNA therapies targeting key genes related to AS pathogenicity have great potential, but their clinical application is limited by nuclease degradation, rapid clearance *in vivo*, low intracellular delivery efficiency and potential immunogenicity. NPs protect siRNA through electrostatic recombination or encapsulation, and integrate cell-penetrating peptides and pH sensitive lipid materials to enhance release efficiency. By using the specific receptors highly expressed within the plaques, specific delivery to the vascular can be achieved, significantly delaying the progression of the plaques (Figure 3) (Khan et al., 2018; Tao et al., 2020; Huang et al., 2022b).

## 7 Summary and prospects

**FIGURE 4:**
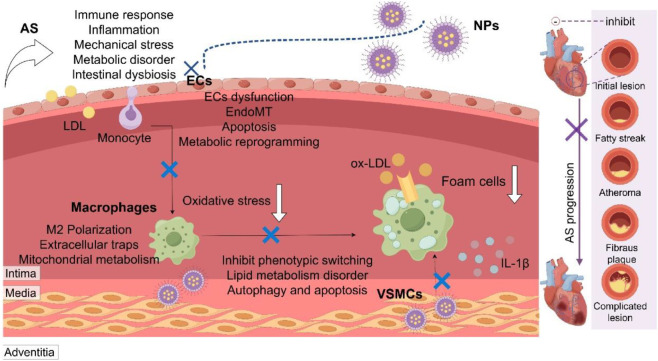
Multifactorial pathogenesis of atherosclerosis and nanoparticle-based targeting strategies. Atherosclerosis (AS) is driven by interconnected pathological factors including immunity, inflammation, mechanical stress, metabolism, and gut dysbiosis. Nanoparticles (NPs) serve as a versatile platform for precise intervention by targeting key pathological processes in major vascular cells: endothelial cells (ECs), macrophages, and vascular smooth muscle cells (VSMCs). LDL, low-density lipoprotein; ox-LDL, oxidized LDL; EndoMT, endothelial-mesenchymal transition.

NPs delivery technology holds great promise for advancing drug and gene therapy in AS. This technology can specifically target and effectively modulate the activity of macrophages and endothelial cells, as well as the local immune microenvironment, thereby stabilizing plaques and enhancing long-term survival rates. Nonetheless, concerns regarding the biosafety of NPs, their distribution, and the stability and controllability of targeted delivery remain critical areas of focus. The development of NPs capable of executing multiple synergistic functions, such as the combined use of encapsulated genes, drugs and the targeted treatment of personalized encapsulated edited gene fragments, presents substantial research value. Furthermore, given the potential for NPs to be engulfed by the immune system prior to reaching their target site, constructing a biological NPs delivery system using the patient’s own macrophages, endothelial cell membranes, exosomes, and platelets to avoid immune rejection is of paramount importance. The mechanisms of AS caused by complex factors such as immunity, inflammation, mechanical stress, metabolism and dysbiosis are different, and the precise treatment guided by NPs remains to be explored. In addition, targeted therapy for the specific pathological phenotypes of ECs, macrophages and SMCs also needs to be studied (Figure 4) (Tang et al., 2024; Packer et al., 2021; Vosoughi et al., 2025; Mo et al., 2024; Li et al., 2025).

It is important to note that upon entering the biological environment, NPs are rapidly coated with a layer of proteins, forming a “protein corona.” This corona can alter the surface properties of NPs, affecting their stability, cellular uptake, and targeting specificity. While it may enhance biocompatibility in some cases, it can also lead to unintended immune recognition and clearance, thereby reducing the accumulation of NPs at the target site. Strategies to mitigate these effects include surface PEGylation, functionalization with stealth coatings, or the use of biomimetic membranes derived from autologous cells to minimize opsonization and improve targeting accuracy (Nienhaus and Nienhaus, 2023; Renzi et al., 2024).

Integrating NPs with existing drugs, including active components from TCM, represents a promising direction for innovation. From a clinical translation perspective, current research predominantly emphasizes animal studies, with a limited number of human clinical trials. Challenges persist in determining the optimal dosage and frequency of NPs administration for clinical application in plaque treatment. Further investigation into the microenvironment of AS plaque pathogenesis and elucidation of the intercellular communication among macrophages, ECs, SMCs, fibroblasts, and foam cells at various disease stages remain pivotal areas of research, concerning the targeting efficacy of NPs. Identifying key genes involved in the phenotypic transformation of foam cells, refining gene editing techniques, and enhancing the ability of NPs to selectively target and accumulate in plaques, thereby restoring vascular homeostasis, hold substantial promise for advancing AS treatment (Mata Corral et al., 2024; Nankivell et al., 2024; Yan et al., 2023).

NPs delivery technology has significant potential for theranostics of AS. Traditional diagnostic and therapeutic methods face challenges in early precise detection and treatment, while NPs offer a breakthrough strategy. Multiple functional NPs, through bionic design and intelligent response mechanisms, significantly enhance the accuracy and efficiency of AS diagnosis and treatment. In terms of targeted delivery systems, the targeting strategy based on pathological characteristics can effectively inhibit lipid deposition and inflammation. In terms of diagnostic integration, NPs can be combined with ultrasound technology to achieve highly sensitive detection and real-time dynamic monitoring of plaque biomarkers. However, the clinical transformation of integrated nano-diagnosis and treatment still faces challenges. NPs may be cleared by the immune system, the metabolic mechanism is not yet fully clarified, and the long-term biological safety needs to be evaluated (Wang et al., 2022; Ramoni et al., 2025).

## 8 Conclusion

NP delivery technology offers innovative strategies for the management of AS. By employing the rational design and construction of NPs delivery systems, efficient and targeted modulation of macrophage and endothelial cell functions can be achieved for purposes such as drug delivery and gene editing. This approach enhances the treatment of plaques and reduces AS related complications, including myocardial and cerebral infarctions. Future research should prioritize the optimization of the safety, controllability, and efficacy of NPs delivery systems, alongside accelerating the translational research process from fundamental science to clinical applications.
